# Evolution of Virulence, Fitness, and Carbapenem Resistance Transmission in ST23 Hypervirulent Klebsiella pneumoniae with the Capsular Polysaccharide Synthesis Gene *wcaJ* Inserted via Insertion Sequence Elements

**DOI:** 10.1128/spectrum.02400-22

**Published:** 2022-10-12

**Authors:** Shuyi Wang, Qi Ding, Yawei Zhang, Anru Zhang, Qi Wang, Ruobing Wang, Xiaojuan Wang, Longyang Jin, Shuai Ma, Hui Wang

**Affiliations:** a Department of Clinical Laboratory, Peking University People’s Hospital, Beijing, China; b Institute of Medical Technology, Peking University Health Science Center, Beijing, China; Brown University

**Keywords:** carbapenem-resistant hypervirulent *Klebsiella pneumoniae*, CR-hvKP, capsule, conjugation, insertion sequences, IS, virulence, *wcaJ*

## Abstract

Carbapenem-resistant hypervirulent Klebsiella pneumoniae (CR-hvKP) is recognized as a threat worldwide, but the mechanisms underlying its emergence remain unclear. As most CR-hvKP isolates are not hypermucoviscous, we speculated that the evolution of the capsule might result in the convergence of carbapenem resistance and hypervirulence. Here, 2,096 K. pneumoniae isolates were retrospectively collected to screen the ST23-K1 clone, and hypervirulence was roughly defined as being highly resistant to serum killing. The effect of *wcaJ* on the capsule, virulence, fitness, and resistance acquisition was further analyzed. The capsule gene *wcaJ*, inserted by IS*Kpn26*/IS*Kpn74*, was identified via whole-genome sequencing in four hvKP, but not hypermucoviscous, isolates. Uronic acid quantitation results revealed that these isolates produced significantly less capsular polysaccharides than NTUH-K2044. A significant increase in capsular production was observed in *wcaJ*-complemented isolates and confirmed by transmission electron microscopy. Further, all *wcaJ*-complemented isolates acquired greater resistance to macrophage phagocytosis, and one representative isolate resulted in a significantly higher mortality rate than the parental isolate in mice, indicating that *wcaJ* inactivation might compromise virulence. However, isolates with *wcaJ* interruption demonstrated a lower fitness cost and a high conjugation frequency of the *bla*_KPC-2_ plasmid, raising concerns about the emergence of carbapenem resistance in hvKP.

**IMPORTANCE** Klebsiella pneumoniae is one of the most common nosocomial pathogens worldwide, and we speculated that the evolution of the capsule might result in the convergence of carbapenem resistance and hypervirulence of K. pneumoniae. The *wcaJ* gene was first reported to be interrupted by insertion sequence elements in ST23-K1 hypervirulent Klebsiella pneumoniae, resulting in little capsule synthesis, which plays an important role in virulence. We examined the effect of *wcaJ* on the capsule, virulence, and fitness. Isolates with *wcaJ* interruption might compromise virulence and demonstrated a lower fitness cost and a high conjugation frequency of the *bla*_KPC-2_ plasmid, highlighting its role as a potential factor facilitating hypervirulence and carbapenem resistance.

## INTRODUCTION

Klebsiella pneumoniae is one of the most common nosocomial pathogens worldwide, posing a serious threat to public health (1). In recent years, two variants of K. pneumoniae, namely, hypervirulent K. pneumoniae (hvKP) and carbapenem-resistant K. pneumoniae (CRKP), have become particularly great concerns, with hvKP causing serious and metastatic infections, such as liver abscess, endophthalmitis, and necrotizing fasciitis, which are associated with high rates of mortality and morbidity in healthy individuals (2). In China, the majority of pyogenic liver abscesses are due to hvKP (3), and most hvKP isolates are susceptible to all available antimicrobials (4). However, the convergence of hypervirulence and carbapenem resistance was identified in K. pneumoniae, with a significant increase in its reported prevalence (5). Carbapenem-resistant hypervirulent K. pneumoniae (CR-hvKP) demonstrates a limited fitness cost compared with the classic CRKP and hvKP strains (6–8), raising concerns about its further dissemination in clinical settings.

Numerous studies have investigated the epidemiology of CR-hvKP, whereas others have dissected the characteristics underlying its antibiotic resistance and virulence (4, 6, 9–11). However, there is still limited knowledge of the molecular mechanisms driving the emergence of CR-hvKP, particularly the element that decreases the fitness cost associated with the cooccurrence of virulence and resistance.

Herein, an analysis of four K. pneumoniae genomes, with a focus on the prevalent clone ST23-K1 hvKP, along with virulence and fitness assays revealed that the *wcaJ* gene is a potential factor that facilitates the combination of hypervirulence and multidrug resistance. The *wcaJ* gene in K. pneumoniae encodes a glycosyltransferase, which is the initiating enzyme of the colanic acid synthesis pathway, and it plays an important role in polysaccharide synthesis (12). Insertion in the *wcaJ* gene via insertion sequence (IS) elements in ST23-K1 hvKP was found to be associated with a low fitness cost and a high conjugation capacity with respect to the *bla*_KPC_ gene, increasing the potential risk of CR-hvKP emergence.

## RESULTS

### Microbiological characteristics of K. pneumoniae isolates.

**FIG 1:**
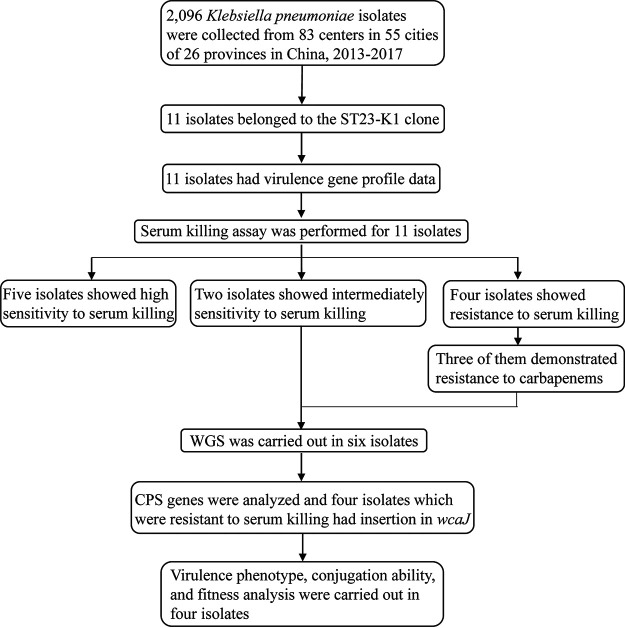
Design and experimental procedures of this study.

In total, 2,096 K. pneumoniae isolates were collected from the China Carbapenem-Resistant Enterobacteriaceae (CRE) Network (13) between 2013 and 2017, with 11 isolates belonging to the ST23-K1 clone (Table 1). Among these, 10 isolates were positive for all critical virulence genes tested, including *iucA*, *iroN*, *rmpA*, and *rmpA2*, indicating that they harbor the pLVPK-like virulence plasmid. Hypermucoviscosity and the ability to defend against serum killing provide valuable information regarding the characterization of hvKP (14). Interestingly, we found that six isolates (designated C228, C400, C1356, C2768, C4120, and C4599) were not sensitive to serum killing and that four of them (C400, C1356, C2768, and C4599) were highly resistant to serum killing (grade 6) but negative for the string test. In addition, four isolates (C228, C400, C1356, and C4599) harbored the *bla*_KPC-2_ carbapenemase gene. Furthermore, the O locus types of these six isolates belonged to the O1/O2v2 serotype, the same as that for NTUH-K2044. The reasons for such phenotypic differences in serum killing and the string test were further investigated in this study. The study design is summarized in Fig. 1.

**TABLE 1:** Microbiological information for ST23-K1 isolates^*a*^

Isolate ID	Collection date (yr/mo/day)	City	K locus	ST	KPC	Serum killing	String test^*b*^	IS element in *wcaJ*	IS*Kpn*26	IS*Kpn*74	*rmpA*	*rmpA2*	O-antigen serotype	O type	*rmpD* type
NTUH-K2044		Taiwan	KL1	ST23		Resistant (grade 6)	+		−	+	+	+	O1/O2v2	O1	2
C1356	2015/2/7	Beijing	KL1	ST23	KPC-2	Resistant (grade 6)	−	IS*Kpn*26	+	+	+	+	O1/O2v2	O1	3
C2768	2017/2/20	Guangzhou	KL1	ST23		Resistant (grade 6)	−	IS*Kpn74*	−	+	+	+	O1/O2v2	O1	3
C400	2015/2/10	Beijing	KL1	ST23	KPC-2	Resistant (grade 6)	−	IS*Kpn*26	+	+	+	+	O1/O2v2	O1	3
C4599	2017/9/12	Kunming	KL1	ST23	KPC-2	Resistant (grade 6)	−	IS*Kpn*26	+	+	+	+	O1/O2v2	O1	
C228	2014/7/4	Nanjing	KL1	ST23	KPC-2	Intermediate (grade 4)	−		−	+	−	+	O1/O2v2	O1	26
C4120	2017/6/9	Zhengzhou	KL1	ST23		Intermediate (grade 4)	+		−	+	+	+	O1/O2v2	O1	2
C1908	2016/12/19	Kunming	KL1	ST23		Sensitive (grade 2)	+		−	+	+	+			
C2362	2017/2/19	Beijing	KL1	ST23		Sensitive (grade 2)	+		−	+	+	+			
C787	2016/3/27	Shenzhen	KL1	ST23	KPC-2	Sensitive (grade 2)	+		−	+	+	+			
C1922	2016/2/10	Tianjin	KL1	ST23		Sensitive (grade 1)	−		−	+	+	+			
C1928	2016/3/1	Tianjin	KL1	ST23		Sensitive (grade 1)	−		−	+	+	+			

a Values for *rmpD* represent *rmpD* typing (15); classification of the O locus was according to Kaptive (16, 37). ST, sequence type. +, positive; −, negative.

b Results for string test.

### Identification of a glycosyltransferase-encoding gene, *wcaJ*, as a potential reason for resistance to serum killing but negative string test results.

**FIG 2:**
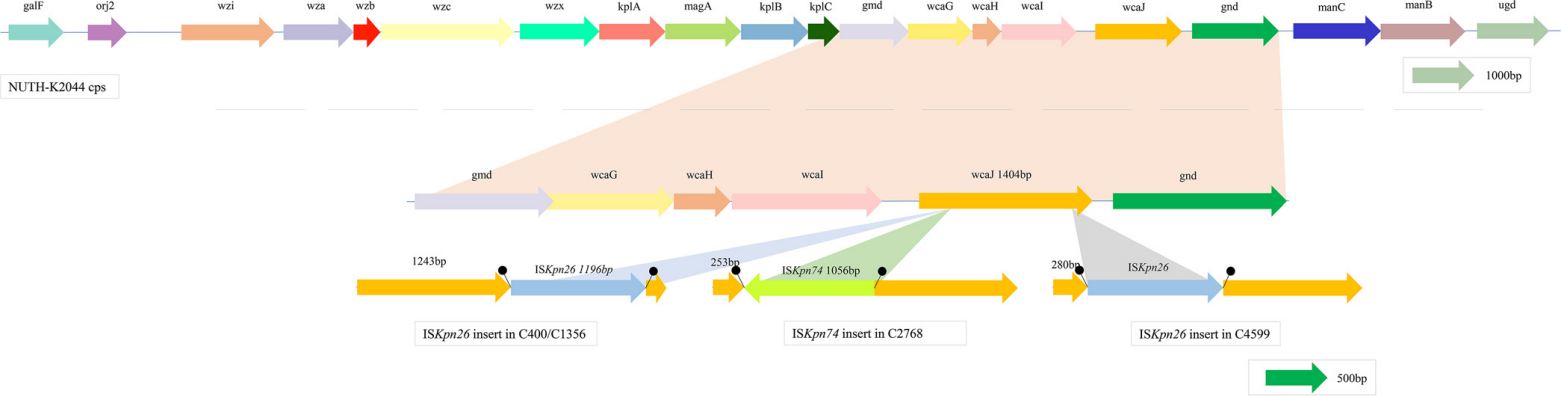
Disruption of *wcaJ* in this study. The Klebsiella pneumoniae K1 capsular polysaccharide biosynthesis gene cluster is shown. The *wcaJ* gene of C400/C1356 was disrupted by the insertion of IS*Kpn26* at nt position 1243; in C4599, IS*Kpn26* was inserted at nt position 280, whereas in the *wcaJ* gene of C2768, IS*Kpn74* was inserted at nt position 253.

**FIG 3:**
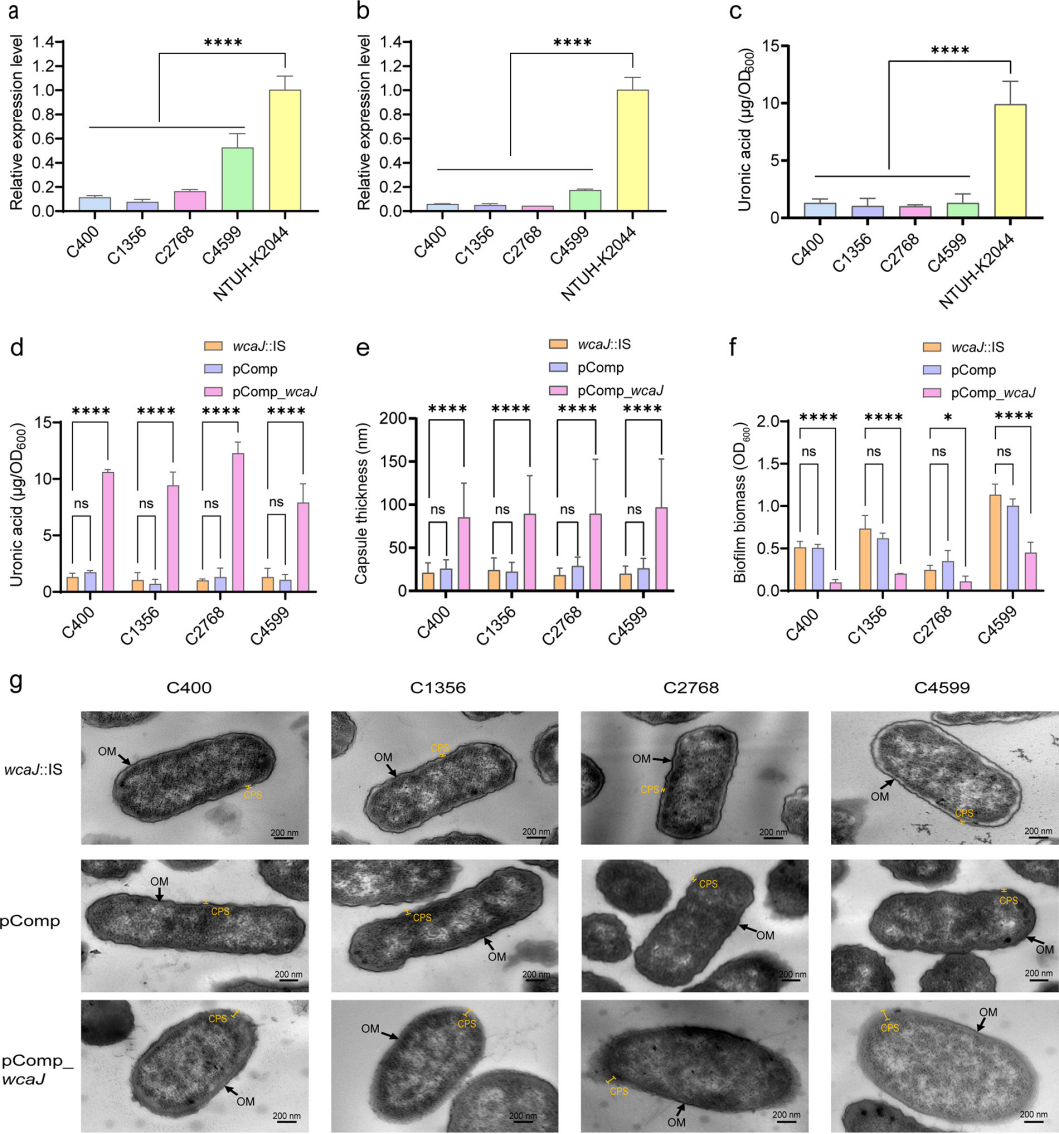
Capsule-related phenotype analysis. (a and b) RT-qPCR analysis of upstream and downstream fragments, respectively, of IS insertion in the *wcaJ* gene region. The NTUH-K2044 isolate was used as a reference. (c) The uronic acid contents of C400, C1356, C2768, and C4599 isolates were compared to that of NTUH-K2044. (d) Uronic acid production was significantly increased in *wcaJ*-complemented isolates compared to that in the parent isolates with IS insertion in *wcaJ* (*wcaJ*::IS). (e) Capsule thickness was determined by measuring five different sites for 10 cells per isolate. The data represent the average of 50 replicates ± standard deviations (SD). (f) Biofilm formation comparison between *wcaJ*::IS and *wcaJ*-complemented counterparts. Six replicates were performed per isolate. (g) Representative TEM images of *wcaJ*::IS, pComp, and *wcaJ*-complemented counterparts. Arrows indicate the outer membrane (OM). Capped lines mark the capsule (CPS). Each data point represents three replicates (*n* = 3), except for those in panels e and f. Data are presented as the mean ± SD. *, *P < *0.05; ****, *P < *0.0001; ns, not significant.

Since capsular polysaccharide synthesis (CPS) is an important factor affecting the hypermucoviscosity phenotype and serum killing (14), we speculated that genes encoding CPS could be altered, giving rise to the phenotypic diversity observed in serum killing and string tests. Four isolates (C400, C1360, C2768, and C4599) were found to have an insertion in the *wcaJ* gene, which was the only mutated CPS gene according to Kleborate, BLAST, and mapping results (15–18) (see Table S1 in the supplemental material). C228 contained an intact *wcaJ* gene but was negative in the string test, which might be due to the absence of the *rmpA* gene (Table 1), despite possessing an intact hypermucoviscosity-related gene, *rmpD* (19). In addition, four isolates with *wcaJ* inserted via an IS element, but not C4599, possessed an intact *rmpD* gene, which did not result in a hypermucoviscosity phenotype. Among these four isolates showing *wcaJ* inactivation, three were CR-hvKP, and IS*Kpn26* was inserted in *wcaJ* in all of these isolates (1,196 bp, IS*5* group, IS*5* family) (5), whereas the other was a hvKP isolate, in which *wcaJ* was found to contain a newly reported IS element, IS*Kpn74* (1,056 bp, IS*903* group, IS*5* family) (20). Figure 2 shows the genomic environment of the wild-type *wcaJ* gene in the reference isolate NTUH-K2044 (21). The *wcaJ* gene was determined to be located in the first contig of the NTUH-K2044 genome (GCF_000009885.1), with a length of 1,404 bp. The IS*Kpn26* insertion site in *wcaJ* was the same in C400 and C1356 (nucleotide [nt] position 1243) but was different in C4599 (nt position 280). Furthermore, IS*Kpn74* was inserted in the *wcaJ* gene of C2768 at nt position 253 (Fig. 2). To confirm the inactivation of the *wcaJ* gene, we designed two pairs of primers to detect *wcaJ* gene expression, and the positions of these are shown in Fig. S1. As expected, compared with that in the reference isolate NTUH-K2044, both fragments showed low expression in all four isolates (Fig. 3a and b) (*P < *0.0001).

### Clinical features of patients infected by isolates with *wcaJ* inactivation.

The medical records of four patients infected by isolates with *wcaJ* inactivation are summarized in Table 2. All isolates were recovered from sputum samples. Notably, patients infected with C400 and C1356 were hospitalized in the same department. All patients suffered from underlying diseases, such as hypertension and heart disease. Except for the patient infected with C4599, most (75%, 3/4) had received antimicrobials 30 days before infection. All of these four patients had pulmonary infections and recovered, except for the patient infected with C2768, who developed a fungal infection and sepsis and finally died.

**TABLE 2:** Clinical characteristics of the four patients infected with *wcaJ*-interrupted isolates

Parameter	Patient 1 (C400)	Patient 2 (C1356)	Patient 3 (C2768)	Patient 4 (C4599)
Age (yr)	52	42	59	62
Gender	Female	Male	Male	Male
City	Beijing	Beijing	Guangdong	Kunming
Type of specimen	Sputum	Sputum	Sputum	Sputum
Ward	Internal medicine	Internal medicine	Internal medicine, ICU^*a*^	Rehabilitation department
Underlying disease(s)	Hypertension	Cerebral hemorrhage, diabetes, hypertension, hemiplegia	Hypertension, coronary heart disease	Hypertension, heart disease, nervous system disease, hemiplegia
Infection type(s)	Pneumonia	Pneumonia	Pulmonary infection, sepsis, fungal infection	Pulmonary infection
Prior antibiotic usage within 30 days	Yes	Yes	Yes	No
Empirical antimicrobial usage	Cefoxitin, levofloxacin	Moxifloxacin, amikacin	Piperacillin/tazobactam, imipenem, teicoplanin	No
Maximum temp (°C)	Unknown	37.4	37	Unknown
WBC^*a*^ (10^9^/L)	Unknown	4.46	16.3	14.79
Therapeutic antimicrobial usage	Unknown	Unknown	Imipenem	Unknown
Outcome	Recovered	Recovered	Died	Recovered

a ICU, intensive care unit; WBC, leukocytes.

### IS element insertion into the *wcaJ* gene among isolates in the public database.

**FIG 4:**
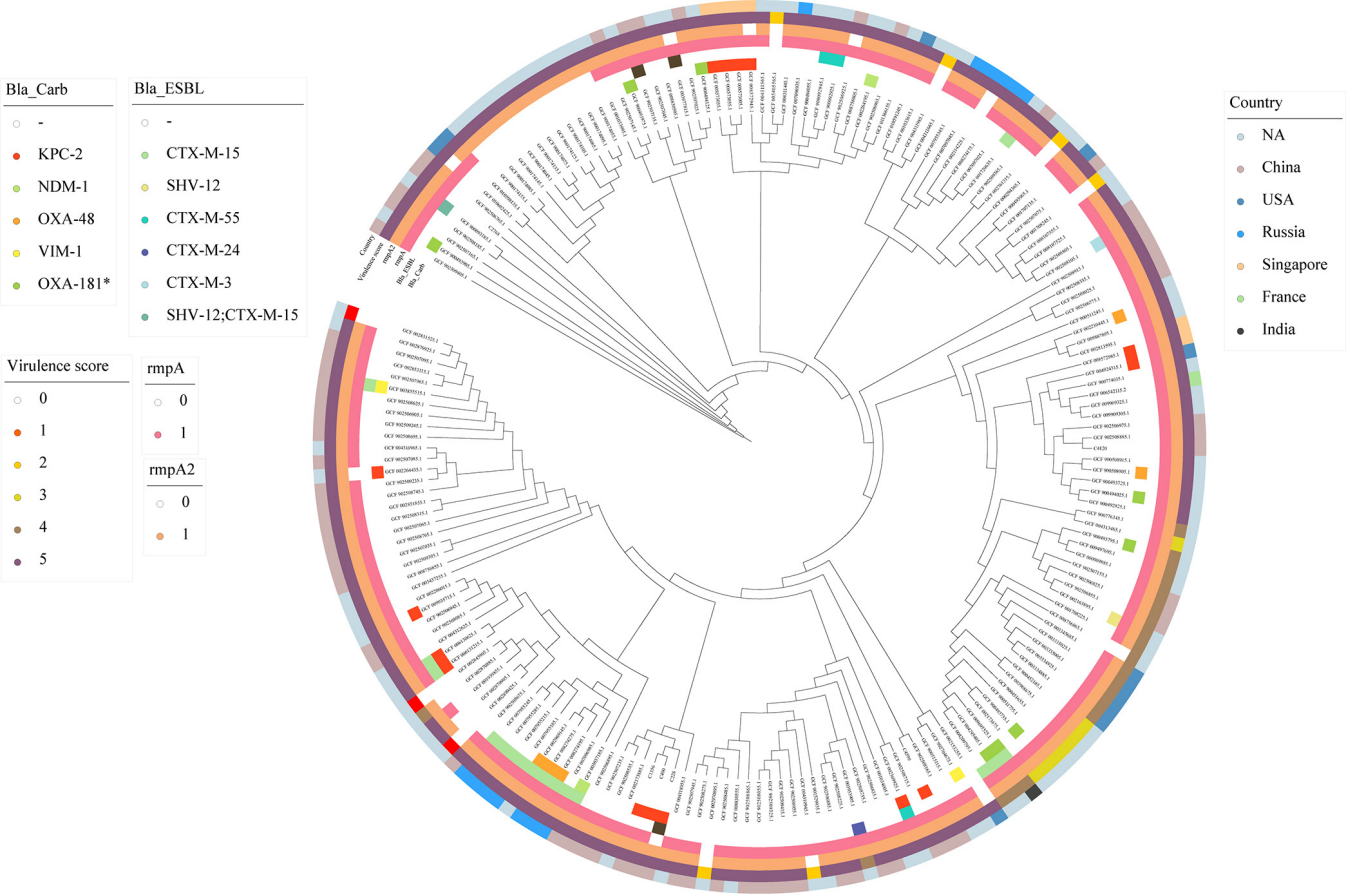
Phylogenetic tree of ST23-K1 isolates. The core genome phylogenetic tree of 191 isolates, including 6 isolates in this study and 185 isolates downloaded from public databases, is shown. On the outside of the tree, the inner two panels provide information about the resistance genes carried by each isolate, including the β-lactamase (Bla), carbapenemase (Carb), and extended-spectrum β-lactamase (ESBL) genes; the three outer subpanels indicate the presence of *rmpA* and *rmpA2* and the virulence score. The outermost panels are colored according to the country of isolates.

To investigate insertions in the *wcaJ* gene among ST23-K1 isolates in public databases, we downloaded 185 K. pneumoniae genomes of the ST23-K1 clone from the NCBI database (Table S1). Among 185 ST23-K1 isolates in the public database, 30 harbored *rmpA* or *rmpA2* deletions and 16 had virulence scores of <4 (Table S1) (15). Isolates with an *rmpA* deletion and those with a virulence score of 4 discernably clustered into different groups in the evolutionary analysis. At the same time, isolates from the same country clustered together (Fig. 4). Furthermore, most ST23-K1 isolates demonstrated the possession of IS*Kpn74* (91/191) (Table 1; Table S2), indicating the potential risk of the insertion of such mobile elements into the *wcaJ* gene. In addition to the four ST23-K1 isolates in this study, we confirmed that another three isolates from the database also had insertions in the *wcaJ* gene (Table S3).

### The *wcaJ* gene plays a critical role in capsule synthesis.

Uronic acid quantitation results revealed that these four isolates produced significantly less capsular polysaccharide than NTUH-K2044 (*P < *0.0001), which was used as a high-capsule-producing reference isolate (Fig. 3c). We hypothesized that the poor capsule synthesis ability of C400, C1356, C2768, and C4599 was due to *wcaJ* inactivation by the IS family. To confirm this, complementation of the wild-type *wcaJ* gene in C400, C1356, C2768, and C4599 isolates was performed. The *wcaJ*-complemented isolates (pComp_*wcaJ*) were positive in the string test (Fig. S2). However, after low-speed centrifugation, the supernatants of two pComp_*wcaJ* isolates (C400 and C1356) remained clear, whereas those of the other two (C2768 and C4599) became significantly turbid with pComp_*wcaJ* plasmid complementation (Fig. S3), indicating hypermucoviscosity. Regarding capsular production, a significant increase was observed in all pComp_*wcaJ* isolates (Fig. 3d) (*P < *0.001). Furthermore, a thicker capsule was detected by transmission electron microscopy (TEM) analysis of the *wcaJ*-complemented isolates (Fig. 3e and g) (*P < *0.0001). Moreover, less biofilm was observed in *wcaJ*-complemented isolates than in clinical isolates with *wcaJ* inactivation (Fig. 3f) (*P < *0.05), suggesting a greater colonization ability among *wcaJ*-interrupted isolates.

### Effect of the *wcaJ* gene on virulence *in vitro* and *in vivo*.

**FIG 5:**
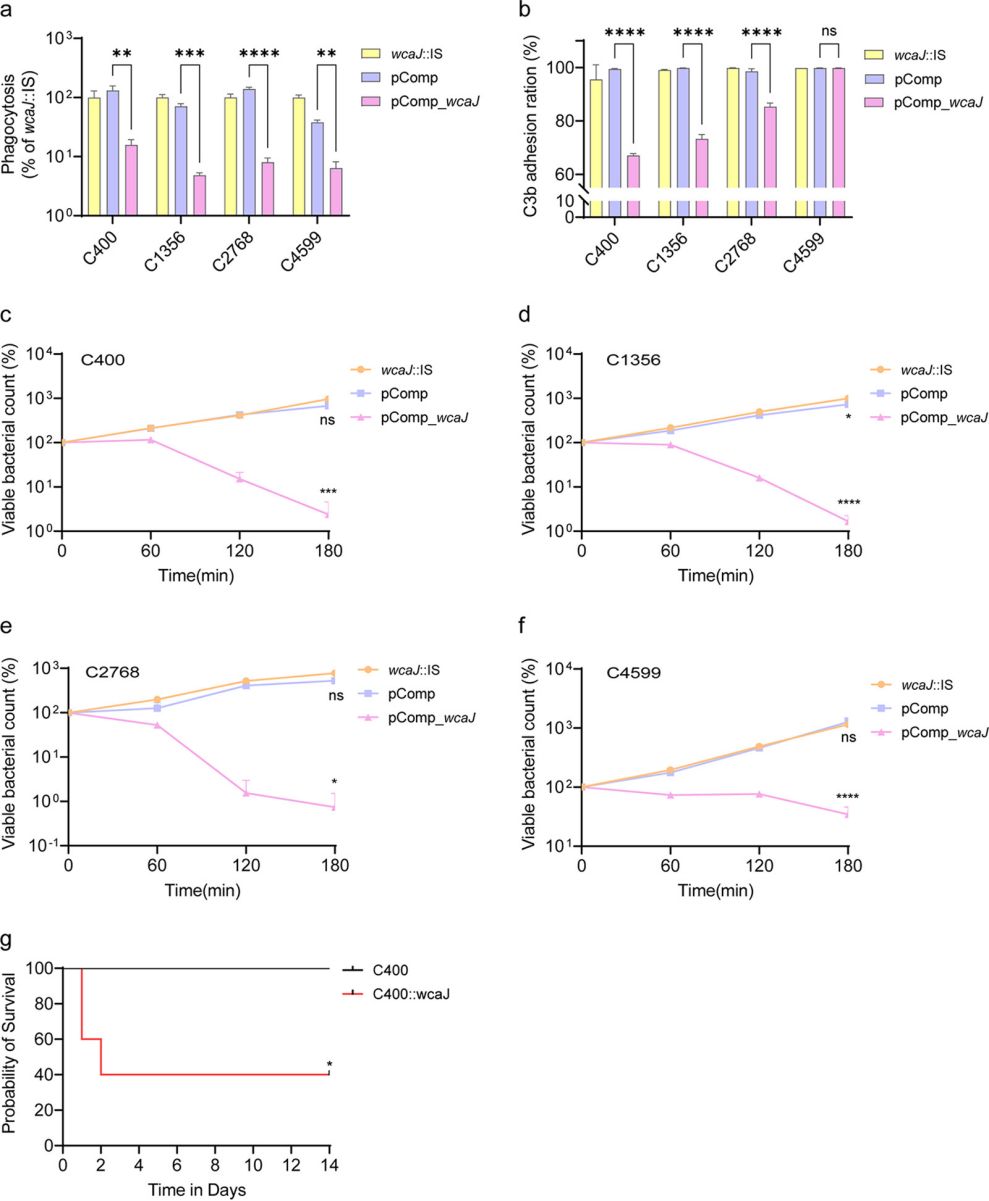
Virulence-related phenotype analysis. (a) Phagocytosis by RAW 264.7 cells. The values shown were calculated as a ratio of the intracellular bacteria to total bacteria and normalized to that of *wcaJ*::IS isolates (*n* = 3). Data were analyzed by one-way ANOVA. Data are presented as the mean ± SD. (b) Flow cytometric analysis of C3b deposition on the surfaces of isolates (*n* = 3). Data were analyzed by one-way ANOVA. Data are presented as the mean ± SD. (c to f) Serum killing results for C400, C1356, C2768, C4599, and their counterparts. Samples were taken at 0, 60, 120, and 180 min, and each isolate was tested three times. Isolates were compared by two-way ANOVA (overall *P < *0.0001), and Tukey’s multiple-comparison test at 180 min showed significant differences. (g) Survival of BALB/c mice. The mice were challenged with 1.96 × 10^6^ CFU of the C400 isolate and 1.97 × 10^6^ CFU of its *wcaJ*-complemented isolate. The mortality of mice was observed over 14 days. The significance was calculated using the log rank (Mantel-Cox) test. *, *P < *0.05; ****, *P < *0.0001; ns, not significant.

To elucidate the effect of the *wcaJ* gene on virulence *in vitro*, serum killing and macrophage phagocytosis assays were performed. All pComp_*wcaJ* isolates acquired greater resistance to macrophage phagocytosis than the parent isolates (Fig. 5a) (*P < *0.01). Intriguingly, in contrast to that observed in the parent isolates that were highly resistant to serum killing (grade 6), pComp_*wcaJ* isolates exhibited increased susceptibility (grade 2 for C400 and C2768 and grade 3 for C1356 and C4599) (Fig. 5c to f). To explore the reason for the inconsistent results between serum killing and macrophage phagocytosis assays, a flow cytometric analysis of C3b deposition on the surface of isolates was conducted. The *wcaJ*-inactivated isolates exhibited high C3b complement adhesion when incubated with serum and fluorescein isothiocyanate (FITC) anti-C3b antibodies, whereas pComp_*wcaJ* isolates showed low C3b complement adhesion, except for C4599 (Fig. 5b). The controversial phenomenon in which *wcaJ*-interrupted isolates with fewer capsules have greater tolerance to serum killing requires further investigation.

A murine infection model was also used to assess virulence *in vivo*, and C400 was selected as a representative isolate. The mortality rate of mice challenged with the C400 *wcaJ*-complemented isolate was significantly higher than that of mice challenged with the parent isolate (Fig. 5g) (*P = *0.049), which indicated that insertion in *wcaJ* might compromise virulence.

### Fitness and conjugation ability of *bla*_KPC-2_ are enhanced by *wcaJ* gene inactivation in hvKP.

**FIG 6:**
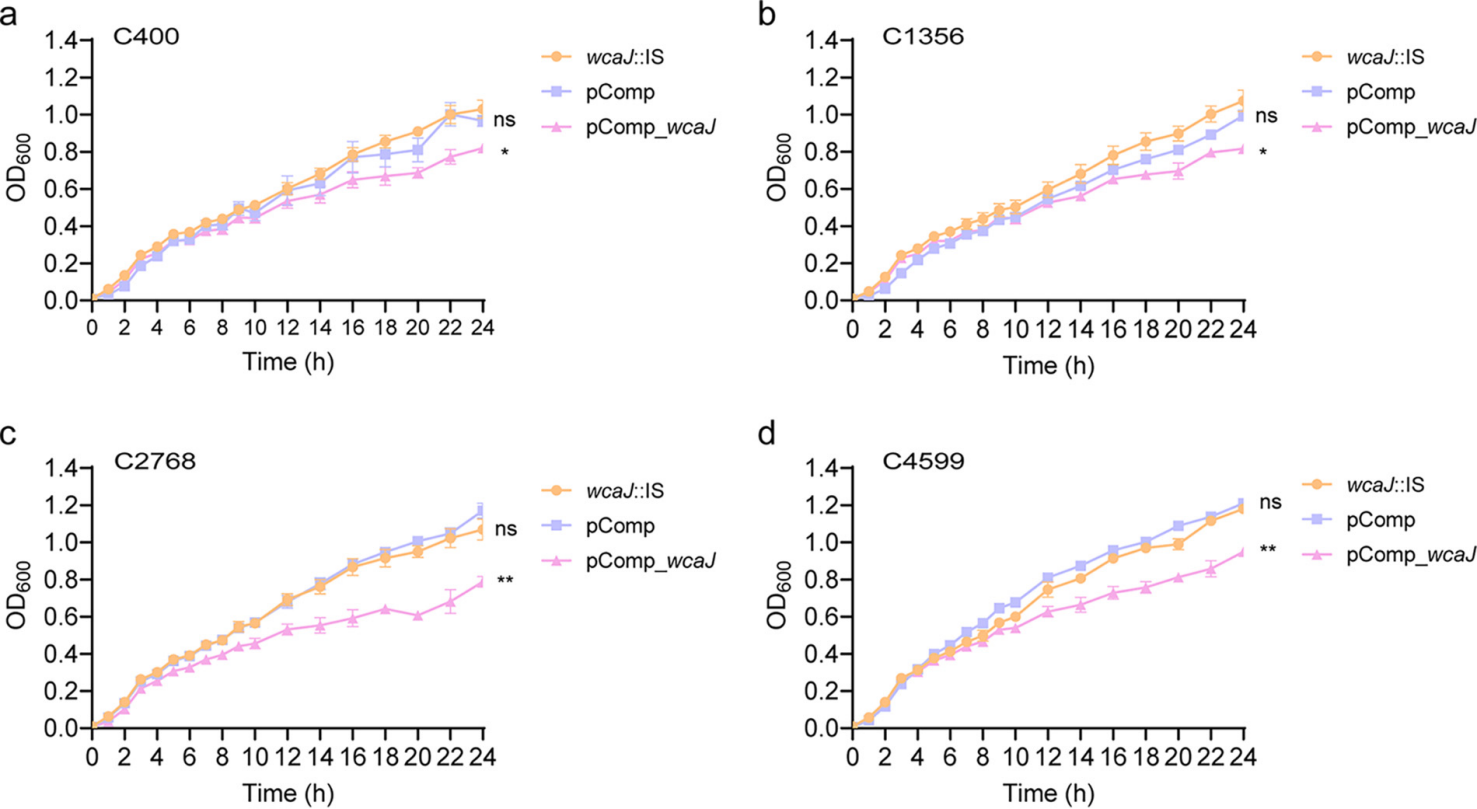
Growth curve of C400, C1356, C2768, and C4599 isolates and their corresponding isogenic derivative strains pComp and pComp_*wcaJ*. (a) C400; (b) C1356; (c) C2768; (d) C4599. Data were analyzed by two-way ANOVA (overall *P < *0.05), and Tukey’s multiple-comparison test at 24 h showed significant differences. *, *P < *0.05; **, *P < *0.01; ns, not significant.

Compared to that in isolates with *wcaJ* interrupted by an IS, a significant fitness cost was observed in *wcaJ*-complemented isolates (Fig. 6) (*P < *0.05). The impact of capsular changes caused by *wcaJ* inactivation on the process of *bla*_KPC-2_ plasmid conjugation was further considered. Since few ST23-K1 isolates with IS elements inserted into the *wcaJ* gene were identified in our database, the KPC plasmids of C400 and C1356 were first eliminated to construct plasmid-curing isogenic derivatives, and conjugation efficiency was then compared between ΔKPC isogenic derivatives and their *wcaJ*-complemented strains. Deletion of the *bla*_KPC-2_ plasmid was confirmed using PCR, and antimicrobial susceptibility testing showed that the susceptibility of carbapenems was restored (Table S4). A higher conjugation frequency (10^−5^) was observed in ΔKPC::pComp isolates than in ΔKPC::pComp_*wcaJ* isolates (conjugation frequency < 10^−8^, *P < *0.01), which showed that complementation of *wcaJ* might prevent KPC conjugation by increasing capsule thickness. In this study, the other two isolates with a *wcaJ* truncation (C4599 and C2768) were not subjected to conjugation assays due to failure in the elimination of the KPC plasmid and the lack of appropriate antibiotic selection markers, respectively.

## DISCUSSION

The *wcaJ* gene encodes a glycosyltransferase; this enzyme loads the first glucose moiety onto the lipid carrier undecaprenyl phosphate, which is responsible for initiating capsule synthesis (22). Previous studies reported that the inactivation of *wcaJ* caused by IS*Kpn26* in the K64 isolate and IS*1* in a K. pneumoniae isolate interrupts capsular polysaccharide synthesis but that *wcaJ*-complemented counterparts show restored CPS production (23, 24), which is consistent with our results. We observed that IS*Kpn26* and IS*Kpn74* were inserted into *wcaJ* in ST23-K1 isolates. Notably, IS*Kpn74* is widespread among ST23-K1 isolates, and IS*Kpn26* is one of the most common mobile elements in *bla*_KPC-2_ plasmids, indicating the potential risks of IS*Kpn26* and IS*Kpn74* transfer.

The capsule is considered an important *in vitro* component related to virulence, and inactivation of the *wcaJ* gene decreases capsule synthesis, thus affecting virulence. Previous studies have proven that Δ*wcaJ* mutants exhibit impaired capsule production and are susceptible to uptake by RAW264.7 macrophages (25), which was also observed for *wcaJ*-interrupted isolates in our study. In addition, respective *wcaJ*-complemented isolates showed restored CPS production and resistance to phagocytosis.

Although the CPS is the main factor protecting K. pneumoniae from the complement system (26), our study showed that *wcaJ* inactivation could increase serum resistance despite producing little CPS. There are two main mechanisms underlying K. pneumoniae tolerance to the bactericidal effect of the complement system (27). One is to avoid complement activation via its thick capsule, whereas the other is to prevent membrane attack complex assembly on the bacterial surface via the long O-polysaccharide side chains of lipopolysaccharide, which prevent C5b-9 formation. Our current findings confirmed an inconsistency between serum resistance and the C3b sedimentation rate, in which the *wcaJ*-inactivated isolates exhibited high C3b complement adhesion and high serum resistance simultaneously. This contradictory phenomenon might reflect failed complement activation, but the exact underlying reason requires further investigation. Furthermore, the bacterial capsule harbors receptors for bacteriophages. A recent study reported that mutations in genes essential for capsule biosynthesis in Acinetobacter baumannii confer resistance to phage adsorption through capsule loss (28). Similarly, the *wcaJ* gene is related to phage sensitivity (29), and its absence might protect isolates, particularly CR-hvKP, from lytic phage predation *in vitro* and *in vivo* (24, 30), thus enhancing their viability.

Consistent with the previous view that the conjugation rate of the resistance plasmid is higher in isolates lacking capsule barriers (31, 32), isolates with lower capsule production owing to *wcaJ* inactivation in this study exhibited greater conjugation efficiency with respect to the *bla*_KPC-2_ plasmid. More alarmingly, isolates with *wcaJ* interrupted by IS elements demonstrated a lower fitness cost than *wcaJ*-complemented isolates. This raises concerns about the emergence and dissemination of carbapenem resistance among ST23-K1 clones of hvKP.

This research, however, is subject to several limitations. First, the complementation experiment was used to evaluate the effects of IS element insertion in *wcaJ*. In fact, the *in situ* replacement approach could be better for demonstrating these effects, as some toxic effects might be caused by *wcaJ* overexpression in Escherichia coli TOP10 (33). Second, as the result in the mouse infection model barely meets statistical significance (five mice per group, *P = *0.049), the virulence assessment of strains *in vivo* can likely be improved by increasing the number of mice per group. In addition, the inclusion of the three other isolates could also contribute to a more comprehensive understanding. Third, the inclusion of more ST23 CR-hvKP isolates in future research will contribute to an expansion of this work.

In conclusion, we report the clinical characteristics and molecular features of four clinical ST23-K1 hvKP isolates with IS elements inserted into the capsule gene *wcaJ*. Further, we dissected the effect of *wcaJ* on capsule synthesis, virulence, and fitness. Our study highlights the role of *wcaJ* as a potential factor that facilitates the combination of hypervirulence and carbapenem resistance, suggesting the urgent need to increase clinical awareness with respect to this matter.

## MATERIALS AND METHODS

### Study design and procedures.

To elucidate the mechanism of carbapenem resistance emergence in ST23-K1 hvKP, we carried out a systemic in-depth study. First, K. pneumoniae isolates, with a focus on CRKP, were retrospectively collected from our national multicenter surveillance network to screen ST23-K1 clones and the hypervirulence phenotype, which was defined as high resistance to serum killing *in vitro*. The capsule is one of the most critical virulence-associated factors of hvKP (34), and our previous work demonstrated that the majority of CR-hvKP strains are negative for the string test, suggesting capsule alterations (data not shown). Thus, we speculated that the evolution of capsules might be a reason for the acquisition of carbapenem resistance in hvKP. For the ST23-K1 isolates with hypervirulence, being intermediate or resistant to serum killing, whole-genome sequencing (WGS) was performed, and genes encoding the capsule were further investigated. We found that *wcaJ* was inserted in the four isolates that were highly resistant to serum killing. To explore the role of *wcaJ* alterations, the effects on capsule, virulence, fitness, and resistance transmission were analyzed.

### Microbiological features.

In total, 2,096 K. pneumoniae isolates were collected from 83 centers in 55 cities in 26 provinces in China between 2013 and 2017. Multilocus sequence typing (MLST) analysis was performed according to the instructions on the MLST website (https://bigsdb.pasteur.fr/klebsiella/), and PCR was used to detect virulence genes (*iucA*, *iroN*, *rmpA*, and *rmpA2*), K serotypes (K1, K2, K5, K20, K54, K57, K47, and K64), and the carbapenemase gene (*bla*_KPC-2_), as previously described (7).

### WGS and analysis.

Six isolates with intermediate or full resistance to serum killing were sequenced using an Illumina NovaSeq system and a HiSeq 2500 system to investigate capsular genes. Reads were assembled and annotated using SPAdes (version 3.13.0) (35) and Prokka (version 1.13.7) (36). The K serotype, MLST, O locus classification, antibiotic resistance genes, and virulence genes were analyzed using Kleborate v2.2.0 and Kaptive v2.0.3 (https://github.com/katholt/Kleborate, https://github.com/katholt/Kaptive) (15, 16, 37). Potential insertion and deletion sites were examined with blastn (version 2.9.0) (18), BWA-MEM (version 0.7.12) (17), and Integrative Genomics Viewer (IGV; version 2.11.4) (38) (see Fig. S4 in the supplemental material) and were further confirmed through PCR and Sanger sequencing. Meanwhile, ISfinder (39) (https://www-is.biotoul.fr/) and blastn were utilized for the identification of IS types.

### Phylogenetic analysis.

To study the distribution of and phylogenetic relationships associated with antibiotic resistance and virulence genes among our isolates and other isolates in the public database, phylogenetic analysis was performed using 191 isolates, including data for 185 isolates downloaded from public databases and six isolates in this study. Kleborate and blastn were used to identify the *wcaJ* gene integrity in the 185 isolates from public databases, and isolates with incomplete whole-genome sequencing data for which the presence of an IS could not be discerned were not included.

### RT-qPCR.

For real-time quantitative PCR (RT-qPCR), total RNA was extracted according to the manufacturer’s instructions (Qiagen, Germany), and reverse transcription was then carried out with 500 ng of RNA using the PrimeScript RT master mix kit (TaKaRa, Japan). Since the *wcaJ* gene was cut into two fragments via IS insertion, two pairs of primers (Table S5) were designed to amplify these fragments, namely, the upstream (156-bp) and downstream (100-bp) regions. The location of these probes is shown in Fig. S1. The reaction mixture was kept at 95°C for 30 s, followed by 40 cycles of 95°C for 5 s and 60°C for 34 s. The ΔΔ*CT* method was used to compare expression differences among isolates. The *rrsE* gene encoding 6-phosphogluconate dehydrogenase and the NTUH-K2044 isolate were used as the internal reference (40) and control isolate, respectively.

### Construction of the *wcaJ*-complemented isolates.

To evaluate the effects of *wcaJ* inactivation caused by IS insertion, we constructed *wcaJ*-complemented isolates. The isolates, primers, and plasmids used are listed in Tables S5 and S6. The ampicillin resistance gene of the pEASY plasmid (TransGen Biotech) was replaced with the tigecycline resistance gene (*tetX4*), resulting in the pEASY-tgc plasmid. The intact *wcaJ* gene from NTUH-K2044 and the *rpsL* promoter from the pCasKP-hph plasmid were then amplified and integrated into the pEASY-tgc plasmid by Gibson assembly, yielding a continuous-expression plasmid (pComp_*wcaJ*). Meanwhile, the pComp plasmid without the *wcaJ* gene was used as a blank control to offset the effect of a foreign plasmid on phenotypic changes.

### Mucoviscosity.

The mucoviscosity of isolates was determined via the string test as previously described (25). Briefly, the string test was performed by stretching the bacterial colony on agar plates by use of a loop. Colonies that stretched at least 5 mm were positive. All experiments were conducted in triplicate. Further, the sedimentation assay was also used to evaluate mucoviscosity (41). After 8 h of incubation, cultures were normalized to 1 OD_600_/mL (where OD_600_ is optical density at 600 nm) and centrifuged at 1,000 × *g* for 5 min. The values of the supernatant were then detected.

### Extraction and quantification of capsule.

Capsular polysaccharides were extracted and quantified as previously described (41, 42). Briefly, 500 μL of the overnight culture was mixed with 100 μL of 1% Zwittergent 3-14 detergent in 100 mM citric acid (pH 2.0) and heated at 50°C for 30 min. Samples were centrifuged for 5 min at 14,000 rpm, and 250 μL of the supernatant was transferred into a new tube and precipitated with 1 mL of absolute ethanol at 4°C for 30 min. After centrifugation for 5 min at 14,000 rpm, the pellets were dried and dissolved in 100 μL of distilled water, and then 600 μL of 12.5 mM sodium tetraborate (Sinopharm Chemical Reagent Co., Ltd) in H_2_SO_4_ was added. Samples were boiled for 5 min and cooled to room temperature for 10 min before the addition of 10 μL of 0.15% 3-phenylphenol (Aladdin). After incubation for 15 min to generate the chromophore, the absorbance at 520 nm was measured. The amounts of CPS were determined from a standard curve of glucuronic acid (Aladdin), and the results were expressed in micrograms per OD_600_ for each culture. All experiments were carried out in triplicate.

### TEM.

To determine capsule thickness, transmission electron microscopy (TEM) (FEI Tecnai Spirit) was performed for isolates C400, C1356, C2768, and C4599 and their corresponding isogenic derivative strains. Overnight cultures were transferred to 1.5-mL polypropylene tubes and centrifuged at 15,000 rpm for 3 min. The pellets were washed with phosphate-buffered saline (PBS) three times and further fixed in 1.5% glutaraldehyde. Image analysis was conducted using ImageJ software.

### Biofilm assay.

Biofilm assays were performed as previously described (43), with some modifications. Briefly, an exponential-phase culture was added to 2.5 mL of LB broth in a polystyrene tube (1:100 dilution) and incubated at 37°C for 48 h. The tubes were washed with PBS, and 4 mL of 0.02% crystal violet was then added. After 15 min of incubation, crystal violet was removed, and each tube was washed three times with PBS. Then, 3 mL of 30% acetic acid was added, and the tube was incubated at room temperature for 30 min. The OD of solubilized crystal violet was measured at 600 nm. All isolates were tested based on six replicates.

### Serum killing assay.

According to our previous study (44), human serum was obtained from 10 healthy volunteers. An inoculum of 25 μL of exponential-phase culture (OD_600_, 0.6) was added to 75 μL of pooled human serum in a 2-mL microcentrifuge tube. Viable counts were checked after incubation for 0, 60, 120, and 180 min at 37°C with 200 rpm of shaking. Each isolate was tested in triplicate. Results were analyzed by comparing the numbers of CFU at each time point and are expressed as highly sensitive (grade 1 or 2), intermediate (grade 3 or 4), or resistant (grade 5 or 6).

### Flow cytometry.

Bacterial flow cytometry was performed as previously described (45). An inoculum of log-phase bacteria (1 × 10^7^ CFU) was incubated with 10% healthy volunteer serum at 37°C for 1 h, and the mixture was incubated with FITC anti-complement C3b antibody (Biolegend) after washing three times with PBS. Samples were analyzed on a FACSCanto system using Diva software (BD Biosciences, San Jose, CA, USA), and at least 200,000 events per sample were acquired for analysis.

### Macrophage phagocytosis assay.

Raw 264.7 murine macrophages were grown in Dulbecco’s modified Eagle’s medium (DMEM) supplemented with 10% fetal bovine serum. Bacterial phagocytosis assays were performed as previously described (46, 47). Macrophages were seeded into 24-well plates and infected at a multiplicity of infection (MOI) of 100 (bacteria/cell). To synchronize the infection, the plates were centrifuged at 200 × *g* for 5 min and incubated at 37°C with 5% CO_2_ for 1 h. Cells were rinsed three times with PBS, followed by the addition of 1 mL of DMEM containing 300 μg/mL gentamicin (except for C2768 and C4599 isolates, which were treated with 300 μg/mL of hygromycin due to gentamicin resistance) to eliminate extracellular bacteria. After 1 h of incubation, cells were rinsed again three times with PBS and lysed with 0.2% Triton X-100 (20-min incubation at room temperature). Tenfold serial dilutions were plated on LB agar plates to determine total CFU counts. The percentage of phagocytosed bacteria per inoculum was calculated and normalized to that of *wacJ*::IS isolates. Three biological replicates were carried out per isolate.

### *In vivo* mouse lethality assay.

The mouse lethality assay was performed as previously described (48). Five 6- to 8-week-old male BALB/c mice, purchased from the Peking University Animal Center, were used for each bacterial concentration in this study. Bacterial isolates were grown overnight in LB broth and subsequently serially diluted to 10^7^ CFU/mL in PBS. A 100-μL bacterial suspension was injected intraperitoneally using a 1-mL sterile syringe. The survival of mice was observed daily for 14 days.

### Growth curve.

Overnight cultures of the *wcaJ*-inactivated and *wcaJ*-complemented K. pneumoniae isolates were diluted to an OD_600_ of 0.01 and grown with shaking (200 rpm) at 37°C for 24 h (49). The OD_600_ was measured every 2 h to obtain the growth curve.

### Generation of the isogenic *bla*_KPC-2_ plasmid deletion mutant.

KPC plasmid curing in CR-hvKP was performed based on clustered regularly interspaced short palindromic repeats (CRISPR), as previously described (50). According to the detailed protocol of the two-plasmid system, pCasKP-pSGKP, for genome editing, we designed a 20-bp spacer using sgRNAcas9 software (51). The spacer was then ligated into the pSGKP-tgc plasmid. Two plasmids, pCasKP-hph and pSGKP-tgc, were electrotransformed into the target isolate one after another for cleavage of the *bla*_KPC-2_ plasmid. Primers used in this step are listed in Table S5. Antibiotics were added at the following concentrations: 100 μg/mL hygromycin and 8 μg/mL tigecycline for both the E. coli and K. pneumoniae isolates. The MICs of the parent strain and the *bla*_KPC_-cured mutants were determined using the Vitek2 automated system (bioMérieux, Marcy l’Etoile, France).

### Conjugation assay of the *bla*_KPC-2_-bearing plasmid.

Conjugation assays were conducted according to previous studies (52). The clinical isolate C1925, which was found to be KPC-2 positive and ciprofloxacin susceptible, was used as a donor, whereas C400ΔKPC::pComp, C400ΔKPC::pComp_*wcaJ*, C1356ΔKPC::pComp, and C1356ΔKPC::pComp_*wcaJ* were used as recipients. The donor and recipient isolates were mixed at a ratio of 1:1 on a 0.22-μm microporous membrane for 24 h. The transconjugants were first selected on LB plates supplemented with meropenem (1 μg/mL) and ciprofloxacin (32 μg/mL) and then confirmed by PCR.

### Statistics.

GraphPad Prism version 9 was used for statistical analysis, including Student’s *t* tests, one-way analysis of variance (ANOVA), two-way ANOVA, and log rank (Mantel-Cox) tests. A *P* of <0.05 was considered statistically significant.

### Ethics.

The Peking University People’s Hospital Institutional Review Board approved this study (no. 2019PHB194-01). Informed consent was obtained from the healthy participants for blood sample collection, whereas patient consent was waived owing to the retrospective nature of this research, and patient data were anonymized. Animal studies were reviewed and approved by the Medical Ethics Committee of Peking University People's Hospital (no. 2019PHE001).

### Data availability.

Data in this study were deposited into NCBI under BioProject accession no. PRJNA791169.
