# Supplementary material for: Evolution of Virulence, Fitness, and Carbapenem Resistance Transmission in ST23 Hypervirulent Klebsiella pneumoniae with the Capsular Polysaccharide Synthesis Gene wcaJ Inserted via Insertion Sequence Elements
Source: Microbiol Spectr. 2022 Oct 12;10(6):e02400-22. doi: 10.1128/spectrum.02400-22 (PMC9769677; doi:10.1128/spectrum.02400-22)
Supplement: Supplemental file 2 — Fig. S1-4. Download spectrum.02400-22-s0002.pdf, PDF file, 0.6 MB [file spectrum.02400-22-s0002.pdf]

Figure S1. The locations of RT-qPCR probes relative to the IS insertion sites.

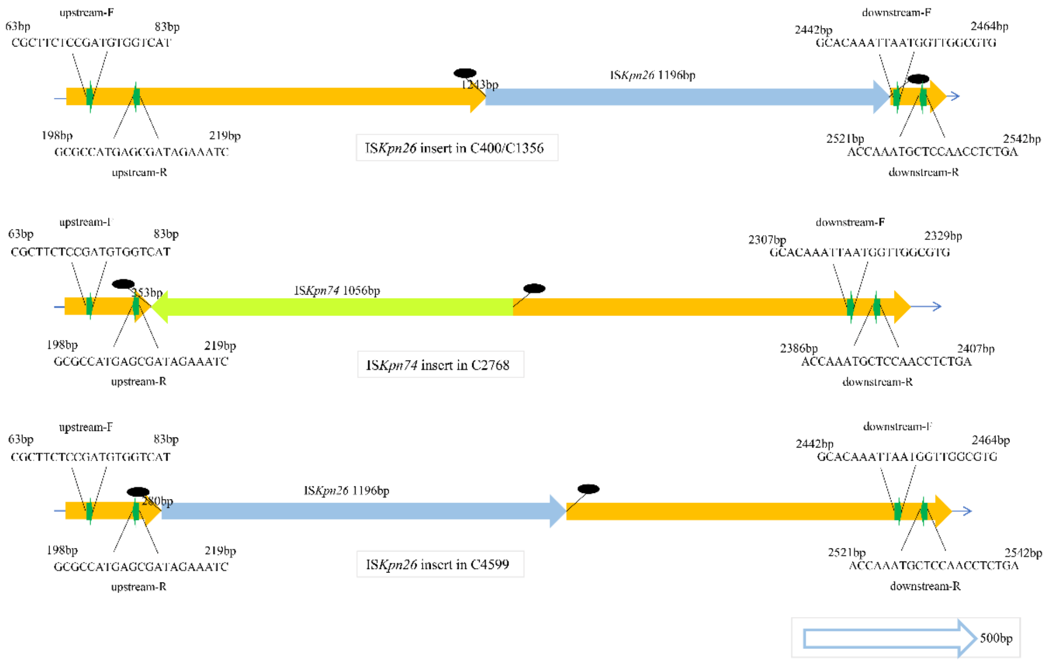

Figure S2. String test were positive in four strains with the pComp_*wcaJ* complementation promoted by *rpsL* promoter.

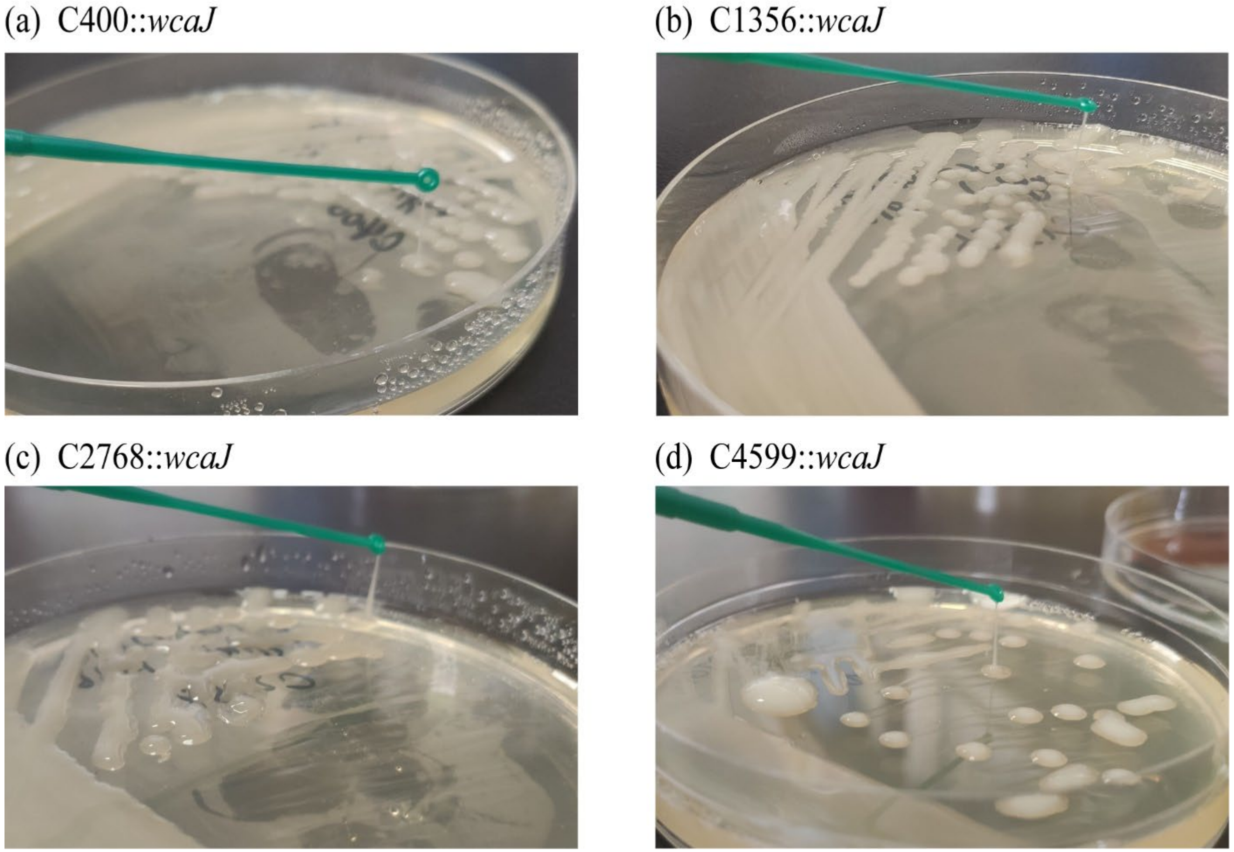

Figure S3. Sedimentation assay results. The supernatant absorbance comparison between *wcaJ*::IS and *wcaJ*-complemented counterparts. Three replicates were performed per isolate.

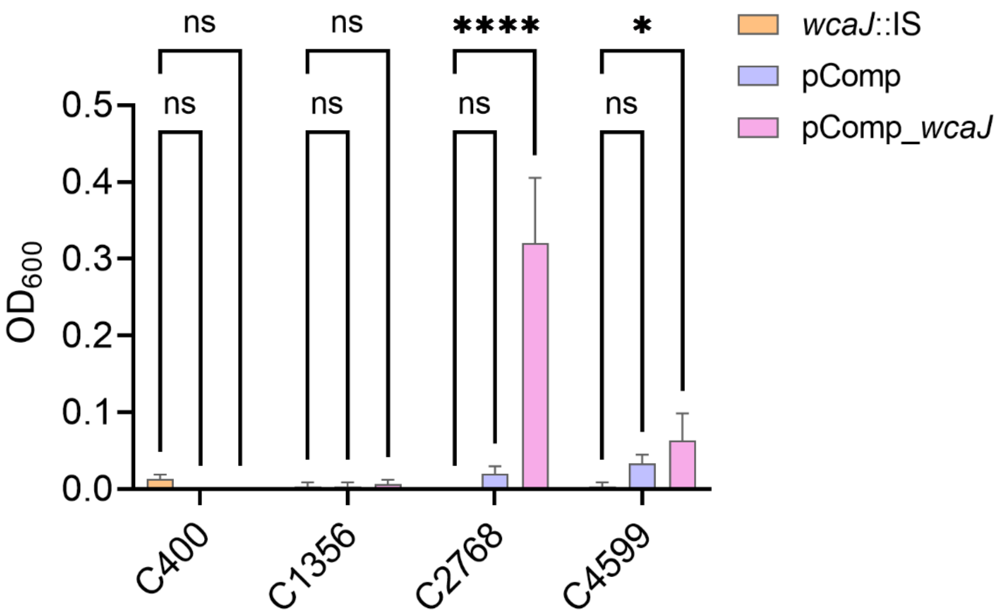

Figure S4. *WcaJ* insertion or deletion sites were examined in the genome data visualization tool Integrative Genomics Viewer (IGV). *WcaJ* is in the first contig of K2044, that is, positions 3527530 to 3528933 on the chromosome. We intercepted the display of the IGV section, and reads of this mapping area were not uniform, indicating that IS might inserted at this position. (a) The mapping result of C400 in IGV. (b) The mapping result of C1356 in IGV. (c) The mapping result of C4599 in IGV. (d) The mapping result of C2768 in IGV.

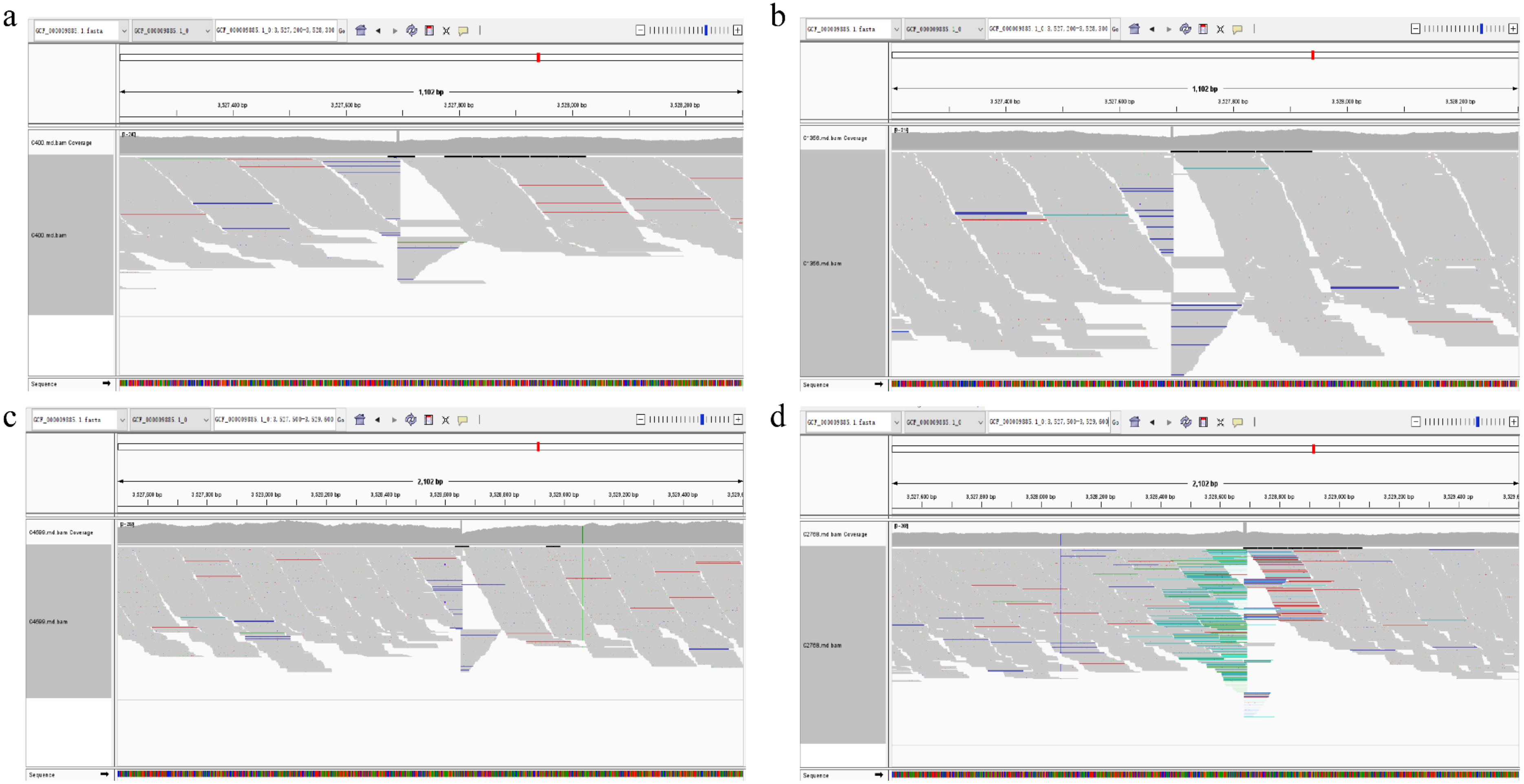
a
b
c
d
1,102 bp
1,102 bp
2,102 bp
2,102 bp
Sequence
